# Fimasartan Ameliorates Nonalcoholic Fatty Liver Disease through PPAR*δ* Regulation in Hyperlipidemic and Hypertensive Conditions

**DOI:** 10.1155/2017/8048720

**Published:** 2017-03-13

**Authors:** Yong-Jik Lee, Yoo-Na Jang, Yoon-Mi Han, Hyun-Min Kim, Jong-Min Jeong, Hong Seog Seo

**Affiliations:** ^1^Cardiovascular Center, Guro Hospital, Korea University, 80 Guro-dong, Guro-gu, Seoul 152-703, Republic of Korea; ^2^The Korea University-Korea Institute of Science and Technology (KU-KIST) Graduate School of Converging Science and Technology, Seoul, Republic of Korea

## Abstract

To investigate the effects of fimasartan on nonalcoholic fatty liver disease in hyperlipidemic and hypertensive conditions, the levels of biomarkers related to fatty acid metabolism were determined in HepG2 and differentiated 3T3-L1 cells treated by high fatty acid and liver and visceral fat tissue samples of spontaneously hypertensive rats (SHRs) given high-fat diet. In HepG2 cells and liver tissues, fimasartan was shown to increase the protein levels of peroxisome proliferator-activated receptor delta (PPAR*δ*), phosphorylated 5′ adenosine monophosphate-activated protein kinase (p-AMPK), phosphorylated acetyl-CoA carboxylase (p-ACC), malonyl-CoA decarboxylase (MCD), medium chain acyl-CoA dehydrogenase (MCAD), and peroxisome proliferator-activated receptor gamma coactivator 1-alpha (PGC-1*α*), and it led to a decrease in the protein levels of 11 beta-hydroxysteroid dehydrogenase 1 (11*β*-HSDH1), fatty acid synthase (FAS), and tumor necrosis factor-alpha (TNF-*α*). Fimasartan decreased lipid contents in HepG2 and differentiated 3T3-L1 cells and liver tissues. In addition, fimasartan increased the adiponectin level in visceral fat tissues. The antiadipogenic effects of fimasartan were offset by PPAR*δ* antagonist (GSK0660). Consequently, fimasartan ameliorates nonalcoholic fatty liver disease mainly through the activation of oxidative metabolism represented by PPAR*δ*-AMPK-PGC-1*α* pathway.

## 1. Introduction

Nonalcoholic fatty liver disease (NAFLD) is a widespread disease defined by excessive fat accumulation in the form of triglycerides (steatosis) in the liver (histologically, over 5% of hepatocytes). In some patients, NAFLD can progress to cirrhosis and further to hepatocarcinoma. NAFLD patients belonging to one subgroup have liver cell injury and inflammation, in addition to excessive fat (steatohepatitis). The latter condition, designated nonalcoholic steatohepatitis (NASH), is virtually indistinguishable histologically from alcoholic steatohepatitis (ASH), and it represents a progressed NAFLD. It was reported that various diseases, such as obesity, diabetes, and hyperlipidemia, can induce the progression of NAFLD and NASH [1–3]. Furthermore, NASH can be used as a representative clinical index together with hypertension, cardiovascular disease, and complications of diabetes [4].

Several compounds such as fenofibrate, a peroxisome proliferator-activated receptor alpha (PPAR*α*) agonist, or statin may help NAFLD and NASH treatments [5–7].

Representative angiotensin II type 1 receptor (AGTR1) blocker, telmisartan, ameliorates NAFLD and NASH through the suppression of macrophage infiltration into the liver, the reduction of adipocyte size, and the elevation of serum adiponectin [8].

Fimasartan (2-n-butyl-5-dimethylaminothiocarbonylmethyl-6-methyl-3-{[2-(1H-tetrazole-5-yl)biphenyl-4-yl]methyl}pyrimidine-4(3H)-one potassium salt trihydrate) represents a nonpeptide angiotensin receptor blocker, with selective AGTR1 blocking effects, which was approved by the Korean Food and Drug Administration in 2010 for the treatment of essential hypertension [9, 10].

Although the effects of other drugs belonging to sartan class, such as telmisartan, on lipid metabolism in liver are relatively well studied, the effects of fimasartan in this context have not been completely elucidated.

In this study, to investigate the potential of fimasartan in NAFLD treatment in hyperlipidemic and hypertensive conditions, the expression levels of various biomarkers related to fatty acid metabolism were determined in HepG2 and differentiated 3T3-L1 cells and liver and visceral fat tissues harvested from spontaneously hypertensive rats (SHRs) given high-fat diet.

In addition, while studies on nuclear receptors such as PPAR*γ* and PPAR*α* in NAFLD have been relatively well studied, researches on the relation between NAFLD and PPAR*δ* are very deficient.

Furthermore, the lowered catabolic metabolism is involved with metabolic diseases such as NAFLD and obesity, and it is well known that PPAR*δ* activates catabolic reactions in cells. So, our study was primarily focused on the relationship between fimasartan and PPAR*δ* in NAFLD. This report presents novel findings showing the relationships between fatty acid metabolism, fatty liver disease, and AGTR1 blocker, fimasartan.

## 2. Materials and Methods

### 2.1. Materials

HepG2 (human hepatocarcinoma cells) and 3T3-L1 (mouse embryonic fibroblasts) cell lines were purchased from Korean Cell Line Bank (Seoul, Korea). Reagents for cell culture such as media, fetal bovine serum (FBS), and antibiotic-antimycotic solution (AA) were bought from WELGENE, Inc. (Daegu, Korea). TRIzol® reagent was purchased from Invitrogen (Grand Island, NY, USA). Power cDNA Synthesis kits, Maxime™ PCR PreMix kits, protein extraction solution, and prestained protein marker were obtained from Intron Biotechnology (Seongnam-si, Gyeonggi-do, Korea). Primers for polymerase chain reaction (PCR) were purchased from Bioneer (Daejeon, Korea). Palmitate, hematoxylin and eosin (H&E), Oil Red O, sodium succinate, nitroblue tetrazolium (NBT), and protease inhibitor cocktail were purchased from Sigma-Aldrich (St. Louis, MO, USA). Kodak GBX developer and fixer reagents were bought from Carestream Health, Inc. (Rochester, NY, USA). Fimasartan was supplied by Boryung Co., Ltd. (Seoul, Korea). Wistar Kyoto (WKY) rats and spontaneously hypertensive rats (SHRs) were purchased from Doo-Yeol Biotech (Seoul, Korea), while the animal diets were purchased from Central Lab. Animal Inc. (Seoul, Korea). Primary antibodies for anti-11 beta-hydroxysteroid dehydrogenase 1 (11*β*-HSDH1), medium chain acyl-CoA dehydrogenase (MCAD), and anti-glyceraldehyde 3-phosphate dehydrogenase (GAPDH) and secondary antibodies were purchased from Santa Cruz Biotechnology, Inc. (Dallas, TX, USA). Primary antibodies against 5′ adenosine monophosphate kinase (AMPK), phosphorylated AMPK (p-AMPK), acetyl-CoA carboxylase (ACC), and phosphorylated ACC (p-ACC) were bought from Cell Signaling Technology, Inc. (Danvers, MA, USA). Primary antibodies for peroxisome proliferator-activated receptor delta (PPAR*δ*), peroxisome proliferator-activated receptor alpha (PPAR*α*), malonyl-CoA decarboxylase (MCD), and peroxisome proliferator-activated receptor gamma coactivator 1-alpha (PGC-1*α*) and rat adiponectin ELISA kit were supplied from Abcam (Cambridge, UK). Primary antibodies for anti-tumor necrosis factor-alpha (TNF*α*) and anti-fatty acid synthase (FAS) were purchased from Novus (Littleton, CO, USA). Chemiluminescent substrate and enhancer solutions were obtained from Bio-Rad (Hercules, CA, USA). Immunohistochemistry reagents were purchased from Vector Laboratories (Burlingame, CA, USA). Reagents to measure the concentrations of total cholesterol, high-density lipoprotein cholesterol (HDL cholesterol), and low-density lipoprotein cholesterol (LDL cholesterol) were bought from Kyowa Medex Co., Ltd. (Tokyo, Japan).

Reagents to estimate the activities for glutamic oxaloacetic transaminase (GOT) and glutamic pyruvic transaminase (GPT) were purchased from DENKA SEIKEN Co., Ltd. (Tokyo, Japan). Rat adenosine triphosphate (ATP) ELISA kit was obtained from MyBioSource (San Diego, CA, USA), and triglyceride colorimetric assay kit was bought from Cayman Chemical Company (Ann Arbor, MI, USA).

### 2.2. Cell Culture

HepG2 cells were cultured in Dulbecco's Modified Eagle's Medium (DMEM) containing 10% FBS and 1% AA solution in 37°C, 5% CO_2_ incubator. The medium was replaced with fresh one every 48–72 h. HepG2 cells between passages 45–56 were plated at a density of 1 × 10^4^ cells per well in 96-well culture dishes or 1 × 10^6^ cells per well in six-well culture plates in DMEM containing 10% FBS and 1% AA. Cells were cultured for 24 to 48 h at 37°C, 5% CO_2_ incubator, and then the media were changed to DMEM containing 1% FBS. Thereafter, cells were treated with high fatty acid (0.1 mmole palmitate), together with fimasartan (0.14 mmole) and PPAR*δ* antagonist, GSK0660 (50 *μ*mol), treatments for 24 h. 3T3-L1 preadipocytes were cultured in DMEM containing 10% calf serum and 1% AA solution at 37°C in a 5% CO_2_ incubator. The medium was replaced every 48–72 h. 3T3-L1 cells between 8–18 passages were plated at a density of 5 × 10^4^ cells per well in 24-well culture dishes in DMEM containing 10% calf serum and 1% AA solution. When 3T3-L1 cells reached confluence, differentiation medium was applied to cells, together with fimasartan (0.14 mmole) and PPAR*δ* antagonist (50 *μ*mol). The differentiation medium contained 0.0125 *μ*mol/mL dexamethasone, 12.5 *μ*mol/mL 3-isobutyl-1-methylxanthine, 10 *μ*g/mL insulin, and 10% FBS. After differentiation for two days, the medium was replaced with insulin medium which contained 10 *μ*g/mL insulin and 10% FBS. After incubation in insulin media for 2–4 days, the medium was changed to maintenance medium which contained only 10% FBS.

### 2.3. Animal Experiment

Seven WKY rats were fed normal control diet containing 10% fat and twenty-four spontaneously hypertensive rats (SHRs) were divided into four groups; group 1 was fed normal control diet (10% fat), group 2 was fed normal control diet and received 10 mg/kg/day of fimasartan, group 3 was fed high-fat diet (60% fat), and group 4 was fed high-fat diet and the animals received fimasartan for 8 weeks. Six-week-old animals were adopted for 7 days, and after 8 weeks all rats were sacrificed during the forenoon of the same day. All animal experiments were in accordance with the Animal Experiment Ethics Guide of Guro Hospital, Korea University. All animal experiments complied with the Korea University Animal Science Rules and Regulations, and the protocols were approved by the Korea University Institutional Animal Care and Use Committee (approval number: KUIACUC-2011-176).

### 2.4. Semiquantitative Reverse Transcription Polymerase Chain Reaction (RT-PCR)

Total RNA was extracted using TRIzol reagent according to the manufacturer's instructions. Complementary DNA was synthesized from total RNA using the Power cDNA Synthesis kit, and polymerase chain reactions for PPAR*δ*, PPAR*α*, PPAR*δ*, and *β*-actin were performed using a PCR PreMix kit. The primer sequences used were as follows: forward 5′-AAGGCCTTCTCCAAGCACAT-3′ and reverse 5′-AAGACGTGCACGCTGATCTC-3′ for human PPAR*δ* (product size, 212 base pairs); forward 5′-CAAACTTGGACCTGAACGAT-3′ and reverse 5′-GAACGGTTTCCTTAGGCTTT-3′ for PPAR*α* (product size, 161 base pairs); forward 5′-TGGAGTTCATGCTTGTGAAG-3′ and reverse 5′-GCATTATGAGACATCCCCAC-3′ for PPAR*γ* (product size, 168 base pairs); forward 5′-GCTTGGTCACTTCGTGGCTA-3′ and reverse 5′-CAAACCGCTTCCAACTCAAA-3′ for *β*-actin (product size, 281 base pairs). The reaction mixture containing cDNA was preheated for 5 minutes at 95°C as an initial denaturation step. Polymerase chain reaction consisted of denaturation step for 20 seconds at 95°C, annealing step for 10 seconds at 55°C, extension step for 30 seconds at 72°C, and final extension step for 5 minutes at 72°C.

### 2.5. Western Blot Analysis

Cells and liver tissues were homogenized in protein extraction solution, and protein amounts of cell and tissue extracts were estimated by the Bradford method. The extracted proteins (10 *μ*g) were loaded onto 10% sodium dodecyl sulfate polyacrylamide gel electrophoresis (SDS-PAGE) gels. Protein blotting to nitrocellulose membranes was performed for 90 min at 100 volts, and the membranes were blocked overnight in 5% skimmed milk solution and washed three times for 10 min with Tris-buffered saline containing 0.05% tween 20 (TBS-T). Primary antibodies were incubated with the membranes at room temperature for 2 h. Dilution conditions for primary antibodies were as follows: PPAR*δ*, PPAR*α*, AMPK, p-AMPK (at Thr172), ACC, p-ACC (at Ser79), MCD, and PGC-1*α* were 1 : 1000, 11*β*-HSDH1 was 1 : 500, TNF*α* was 1 : 500, and MCAD, FAS, and GAPDH were 1 : 200. After the additional washing three times for 10 min, with TBS-T, the membranes were incubated with horseradish peroxidase conjugated secondary antibodies at room temperature for 1 hr. Dilution conditions for secondary antibodies were as follows: anti-rabbit IgG antibodies for PPAR*δ*, AMPK, p-AMPK, ACC, p-ACC, MCD, MCAD, and PGC-1*α* were 1 : 4000, anti-rabbit IgG antibodies for FAS and 11*β*-HSDH1 were 1 : 5000, anti-rabbit IgG antibody for TNF*α* was 1 : 3000, and anti-goat IgG antibody for GAPDH was 1 : 5000. Afterward, the membranes were washed three times for 10 min with TBS-T and once with TBS for 10 min, and the membranes were treated with chemiluminescent substrate and enhancer solutions. Images were obtained manually using developer and fixer reagents, and the results were analyzed by ImageJ program.

### 2.6. The Levels of Total Cholesterol, HDL Cholesterol, LDL Cholesterol, GOT, and GPT in Serum

The levels of total cholesterol, HDL cholesterol, LDL cholesterol, GOT, and GPT in serum samples were estimated by TOSHIBA TBA-2000FR (Toshiba Medical Systems Corporation, Tochigi, Japan) according to manufacturer's instructions in the Department of Laboratory Medicine (Diagnostic Tests), Korea University, Guro Hospital (Seoul, Korea).

### 2.7. Immunohistochemistry

Tissue slides were sequentially soaked in xylene and 100% to 75% graded ethanol solutions to remove the paraffin and rehydrate them. Deparaffinized and rehydrated slides were incubated with 3% H_2_O_2_ solution for 10 min, washed, and blocked using normal serum solution for 1 hr. Afterward, the slides were treated with primary antibody for 1 hr and washed with TBS-T. Following this, slides were incubated with secondary antibody for 30 min and washed with TBS-T, and the premixed VECTASTAIN ABC solution was incubated with the slides for 30 min. The slides were washed with TBS-T and incubated with 3,3′-diaminobenzidine (DAB) substrate solution until the development of color. Furthermore, the slides were washed with tap water for 5 min, counterstained with hematoxylin, washed again with tap water, air-dried, and finally mounted.

### 2.8. Hematoxylin and Eosin (H&E) Staining

Liver and visceral fat tissues, isolated from all rats, were harvested and fixed in 4% paraformaldehyde. The samples were embedded in paraffin and cut into slices (4-5 *μ*m thick) using microtome, and the slices were stained with H&E. They were further visualized using an optical microscope (Olympus BX51, Tokyo, Japan) and photographed.

### 2.9. Oil Red O Staining

Cell culture medium was completely removed, and the cells were rinsed with phosphate buffered saline (PBS). Afterward, PBS was aspirated completely, 10% formaldehyde solution was added to the cells, and they were incubated for 30 min at room temperature. After removing formaldehyde solution, the fixed cells were gently washed with PBS. The Oil Red O solution was added to the wells, and the samples were incubated for 60 min at room temperature, followed by washing with PBS and photographing using an optical microscope (Olympus BX51). Oil Red O dye was eluted by isopropyl alcohol and the absorbance was measured using SpectraMax Plus 384 Microplate Reader (Molecular Devices LLC., Sunnyvale, CA, USA) at 530 nm.

### 2.10. Adiponectin Concentration in Visceral Fat

Visceral fat tissues of 50 *μ*g were homogenized in protein extraction solution of 500 *μ*L. The extracts were incubated in −20°C for 20 min and then were centrifuged at 13,000 rpm for 5 min at 4°C. The supernatants were assayed immediately. The adiponectin standard or sample of 50 *μ*L was added to each well of 96-well plates, and after covering wells with a sealing tape, it was incubated for 1 hr at room temperature. After washing of 5 times with 200 *μ*L of 1x wash buffer, 50 *μ*L of 1x biotinylated adiponectin antibody was added to each well, and it was incubated for 1 hr. After 5 times washing with 200 *μ*L of 1x wash buffer, 50 *μ*L of 1x streptavidin-peroxidase conjugate was added to each well, and it was incubated for 30 min. After 5 times washing with 200 *μ*L of 1x wash buffer, 50 *μ*L of chromogen substrate was added to each well, and it was incubated till the optimal blue color density develops. 50 *μ*L of stop solution was added to each well, and then the absorbance was estimated with a microplate reader at a wavelength of 450 nm.

Adiponectin concentration in the samples was determined by interpolation from a standard curve prepared with standard samples supplied by the manufacturer and expressed in ng/mL.

### 2.11. Triglyceride and ATP Concentrations in Liver

Enzymatic colorimetric assay kit was used to measure triglyceride concentrations in the liver tissues, according to the manufacturer's instructions. Sample preparation procedures were as follows. Liver tissue was rinsed in ice-cold PBS to remove excess blood. The 300 mg of minced liver tissue was homogenized in 2 mL of the diluted standard diluent. The extract was centrifuged at 10,000 ×g for 10 min at 4°C, and then the supernatant was transferred to another tube. The supernatant sample was diluted at the ratio of 1 : 5 before use. Standard and sample solutions (10 *μ*L) were added to the wells, and the diluted enzyme buffer solution (150 *μ*L) was added to each well. Afterward, the plate was carefully shaken for a few seconds, covered with a sealing tape, and incubated for 15 min at room temperature. The absorbance at 540 nm was measured using a microplate reader. Triglyceride concentrations in the samples were determined by interpolation from a standard curve prepared using the standard solutions and expressed in mg/dL.

ATP concentration in the liver tissues was analyzed using ELISA kit, according to the manufacturer's instructions. The excess blood of liver tissues was washed out by ice-cold PBS. The 300 mg of minced liver tissues was homogenized in 500 *μ*L of PBS. The extracts were subjected to two freeze-thaw cycles to further break the cell membranes. The homogenates were centrifuged for 15 minutes at 1500 ×g, and then the supernatants were assayed immediately. Briefly, standards and samples (100 *μ*L) were added to the wells, followed by the addition of conjugate solution (50 *μ*L), and the plate was covered and incubated for 1 h at 37°C. After washing the plate, a substrate solution (100 *μ*L) was added to each well, and the plate was covered and incubated for 10 min at 37°C. Afterward, a stop solution was added, and the absorbance was measured at 450 nm using a microplate reader. ATP concentration in the samples was determined by interpolation from a standard curve prepared with standard samples supplied by the manufacturer and expressed in ng/mL.

### 2.12. Succinate Dehydrogenase (SDH) Activity Assay

Liver tissues were homogenized in PBS containing 1% protease inhibitor cocktail. Homogenized extracts were centrifuged at 13,000 rpm for 5 min at 4°C, and the supernatants were transferred to new tubes. Incubation solution consisted of 25 *μ*L of 1 mole phosphate buffer, 125 *μ*L of 0.2 mole sodium succinate, 25 *μ*L of 10 mg/mL nitroblue tetrazolium (NBT), and 235 *μ*L of distilled water, per reaction, and the samples were incubated with it for 20 min at 37°C. Enzyme solution (90 *μ*L) was added to 410 *μ*L of prewarmed incubation solution, and the reaction mixture was incubated at 37°C. Following the reaction, the absorbance was measured at 550 nm.

### 2.13. Statistical Analysis

Data are presented as the mean ± SEM (standard error of the means). Statistically significant differences between two groups were calculated by the unpaired* t*-test, and one-way ANOVA test was used to compare means of three or more groups. When the *p* value was less than 0.05, it was considered significant.

## 3. Results

### 3.1. RT-PCR Results for PPAR*δ*, PPAR*α*, and PPAR*δ* in HepG2 Cells Treated with Palmitate and Fimasartan

**Figure 1:**
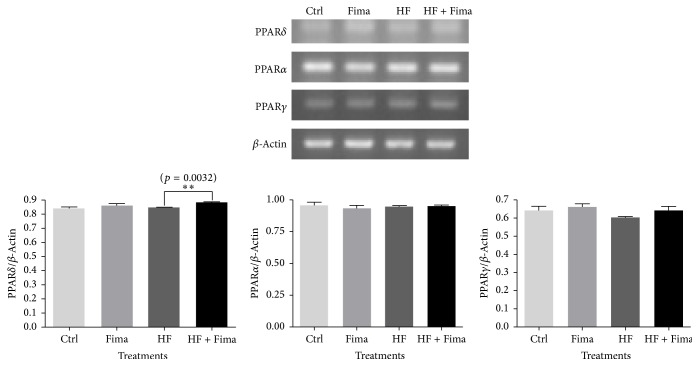
Peroxisome proliferator-activated receptor delta (PPAR*δ*), peroxisome proliferator-activated receptor alpha (PPAR*α*), and peroxisome proliferator-activated receptor gamma (PPAR*γ*) mRNA expression in HepG2 cells treated with high fatty acid and fimasartan. Total RNA was extracted from HepG2 cells treated with high fatty acid and fimasartan, and then complementary DNA (cDNA) was synthesized from the total RNA. PCR reactions for PPAR*δ*, PPAR*α*, and PPAR*γ* were performed. The density of bands on agarose gel was analyzed with ImageJ program. The results are expressed as means ± SEM (*n* = 3). Values were statistically analyzed by unpaired* t*-test. All experiments were repeated three times.

Fimasartan treatment increased the mRNA expression level of PPAR*δ* compared to high fatty acid control. But the mRNA levels of PPAR*α* and PPAR*γ* were not changed by fimasartan in both normal and high fatty acid conditions (Figure 1).

### 3.2. Western Blot Analyses for PPAR*δ*, AMPK, ACC, FAS, 11*β*-HSDH1, TNF-*α*, MCD, MCAD, and PGC-1*α* in HepG2 Cells Treated with Palmitate and Fimasartan

PPAR*δ* is ubiquitously expressed in many tissues such as liver, heart, colon, and skeletal muscle, and its functions may be related to several chronic diseases, including diabetes, obesity, atherosclerosis, and cancer [11, 12].

**Figure 2:**
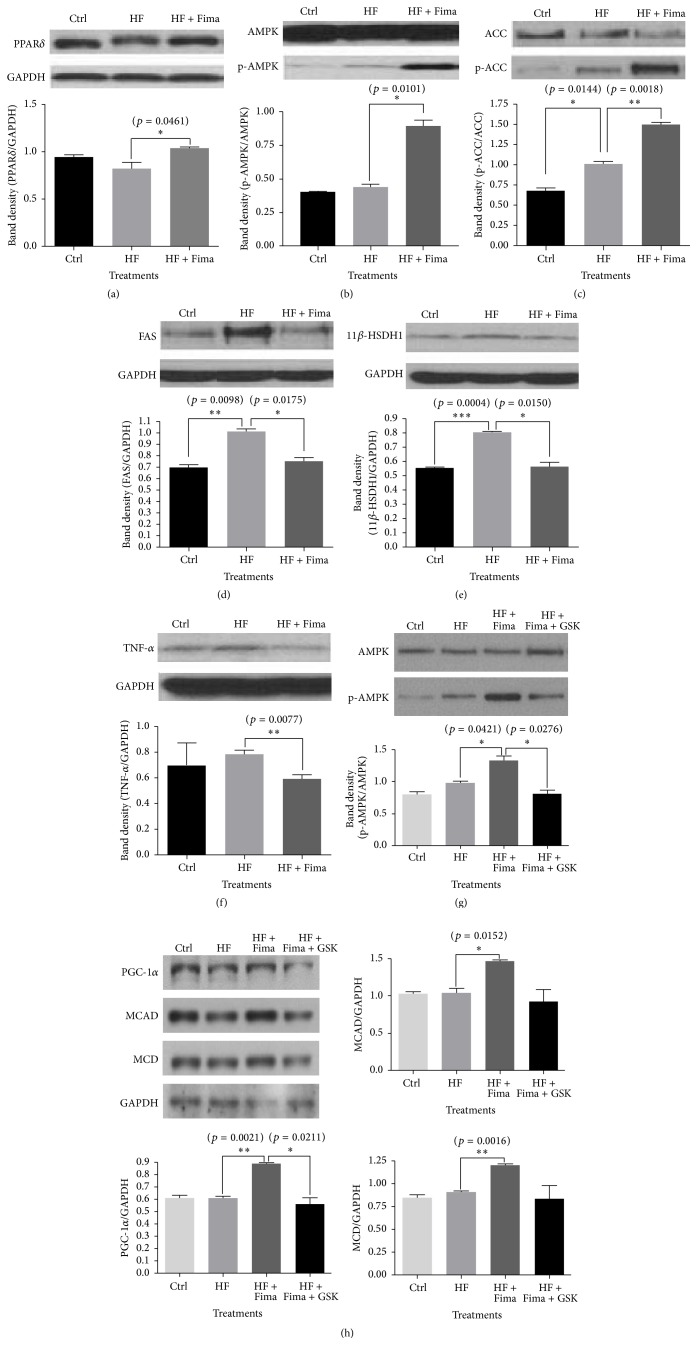
Peroxisome proliferator-activated receptor delta (PPAR*δ*), 5′ adenosine monophosphate-activated protein kinase (AMPK), 11 beta-hydroxysteroid dehydrogenase type 1 (11*β*-HSDH1), tumor necrosis factor-alpha (TNF-*α*), peroxisome proliferator-activated receptor gamma coactivator 1-alpha (PGC-1*α*), medium chain acyl-CoA dehydrogenase (MCAD), and malonyl-CoA decarboxylase (MCD) protein expression in HepG2 cells treated with high fatty acid and fimasartan. Protein extracts were electrophoresed in 10% polyacrylamide gel and blotted to nitrocellulose membrane. The nitrocellulose membrane was bound with primary and secondary antibodies sequentially, and then the chemiluminescent signal was exposed to X-ray film. The density of bands on X-ray film was analyzed with ImageJ program. The results are expressed as means ± SEM (*n* = 3). Values were statistically analyzed by unpaired* t*-test. All experiments were repeated three times.

HepG2 cells treated with palmitate showed a decreased trend in PPAR*δ* level, in comparison with that in the control; however, the level was elevated by fimasartan cotreatment (Figure 2(a)).

AMPK is involved in many pathophysiological reactions such as diabetes, obesity, atherosclerosis, dyslipidemia, inflammation, and cancer, as well as the regulation of cellular energy homeostasis [13, 14]. Phosphorylated AMPK is the active form of AMPK, and the level of phosphorylated AMPK was increased more by fimasartan compared to palmitate only treatment group in HepG2 cells (Figure 2(b)).

ACC catalyzes the production of malonyl-CoA from acetyl-CoA, and then malonyl-CoA is transported to the fatty acid synthesis pathway. AMPK regulates fatty acid synthetic reaction via ACC, and the phosphorylated form of this protein is inactive. Fimasartan significantly increased the protein level of phosphorylated ACC compared to palmitate treatment control in HepG2 cells (Figure 2(c)).

The main function of FAS is to catalyze the synthesis of palmitate from acetyl-CoA and malonyl-CoA in the presence of NADPH [15].

The increase of FAS protein expression induced by palmitate was returned to the normal level by fimasartan treatment (Figure 2(d)).

Cortisol is released under the control of the hypothalamus-pituitary gland-adrenal gland (HPA) axis. In response to stress, corticotropin-releasing hormone (CRH) is secreted from the hypothalamus, the CRH stimulates the release of adrenocorticotropic hormone (ACTH) from the anterior pituitary, and then ACTH induces the secretion of cortisol from adrenal cortex. The cortisol concentration is tightly regulated by 11 *β*-hydroxysteroid dehydrogenase types 1 and 2. The 11 *β*-hydroxysteroid dehydrogenase type 1, a nicotinamide adenine dinucleotide phosphate (NADPH) dependent reductase, produces cortisol from cortisone, while the type 2 isozyme, NAD-dependent dehydrogenase, converts cortisol to cortisone [16]. This also occurs in local tissue, including liver and adipose tissue. The expression of 11*β*-HSDH1 protein was increased by palmitate treatment, but it was inhibited after fimasartan treatment (Figure 2(e)), which has been demonstrated here for the first time.

TNF-*α* is a representing inflammatory cytokine; in our study, its expression showed an increased trend by palmitate. However, fimasartan treatment inhibited the expression of TNF-*α* to below the control level in HepG2 cells (Figure 2(f)).

When the malonyl-CoA decarboxylase (MCD) is activated by AMPK, it inhibits fatty acid synthesis [17]. Medium chain acyl-CoA dehydrogenase (MCAD) is involved in fatty acid oxidation, and it is regulated by AMPK [18]. The protein levels for MCD and MCAD were more increased by fimasartan than high fatty acid treatment group, and PPAR*δ* antagonist (GSK0660) treatment showed the trend to lower these expressions (Figure 2(h)).

PPAR*δ* antagonist, GSK0660, led to a decrease in p-AMPK and PGC-1*α* expression levels, which were previously increased by fimasartan treatment. This suggests that PPAR*δ* may regulate AMPK and PGC-1*α* expression (Figures 2(g) and 2(h)).

### 3.3. Oil Red O Staining of HepG2 and Differentiated 3T3-L1 Cells Treated with Palmitate, Fimasartan, and PPAR*δ* Antagonist, GSK0660

**Figure 3:**
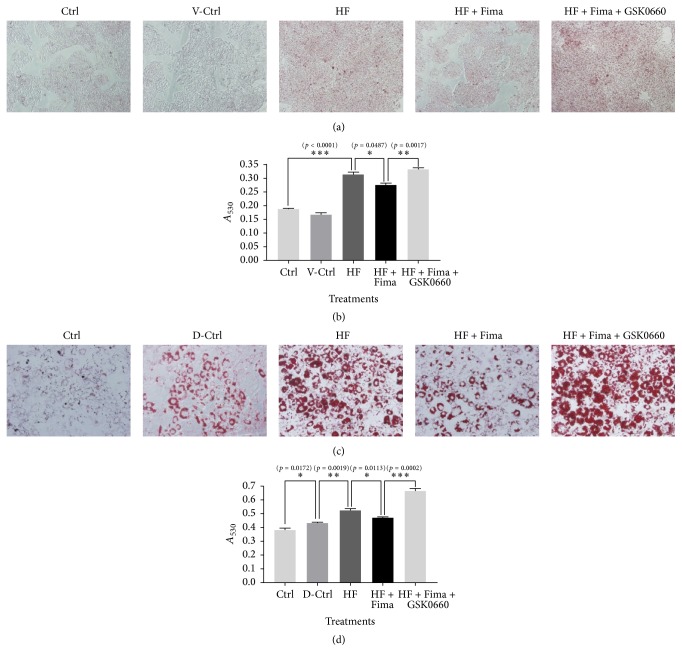
Oil Red O staining in HepG2 and differentiated 3T3-L1 cells treated by palmitate, fimasartan, and GSK0660. (a) Photographs for Oil Red O staining in HepG2 cells treated with palmitate, fimasartan, and GSK0660. The images were taken at 200x magnification. (c) Photographs for Oil Red O staining in differentiated 3T3-L1 cells treated with palmitate, fimasartan, and GSK0660. The images were taken at 400x magnification. ((b) and (d)) After Oil Red O staining, the absorbance for Oil Red O dye eluted by isopropyl alcohol was estimated at 530 nm wavelength. The results are expressed as means ± SEM (*n* = 6). Values were statistically analyzed by unpaired* t*-test. All experiments were repeated three times.

HepG2 and differentiated 3T3-L1 cells treated with palmitate showed an increased intensity of Oil Red O staining compared to the nontreated cells. However, following the treatment with fimasartan, the staining intensity decreased (Figures 3(a) and 3(c)). The absorbance measurements confirmed these results (Figures 3(b) and 3(d)).

### 3.4. Concentrations of Total Cholesterol, HDL Cholesterol, LDL Cholesterol, GOT, and GPT in Serum Samples Isolated from WKY Rats and SHRs

**Figure 4:**
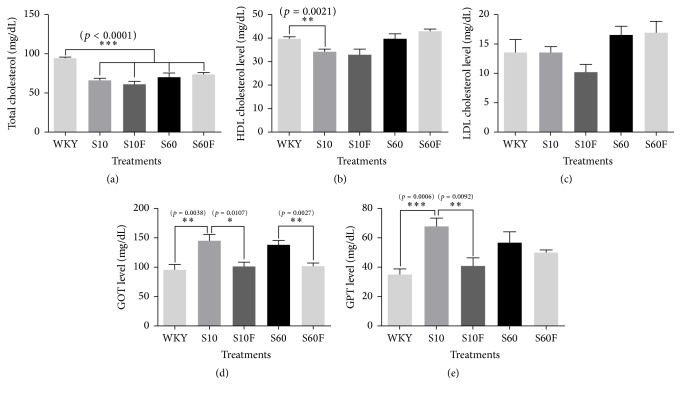
Levels of total cholesterol, high-density lipoprotein cholesterol (HDL cholesterol), low-density lipoprotein cholesterol (LDL cholesterol), glutamic oxaloacetic transaminase (GOT), and glutamic pyruvic transaminase (GPT) in serum of WKY rats and SHRs given high-fat diet and fimasartan. The concentrations of total cholesterol, HDL cholesterol, LDL cholesterol, GOT, and GPT in serum were estimated with TOSHIBA TBA-2000FR according to manufacturer's instructions. The results are expressed as means ± SEM (*n* = 6). Values were statistically analyzed by unpaired* t*-test or one-way ANOVA. All experiments were repeated three times. Meaning of indications: WKY means normal control rat given normal diet containing 10% fat, S10 means SHRs given normal diet containing 10% fat, S10F means SHRs given normal diet containing 10% fat plus fimasartan, S60 means SHRs given high-fat diet containing 60% fat, and S60F means SHRs given high-fat diet containing 60% fat plus fimasartan.

The concentrations of total cholesterol, HDL cholesterol, and LDL cholesterol were not significantly changed with fimasartan treatment (Figures 4(a)–4(c)). But, in the case of GOT, the concentration was significantly elevated in serum samples of SHRs compared to WKY rats, and the increased concentration of GOT in serum of SHRs was normalized to the level of WKY rats by fimasartan treatment (Figure 4(d)). GPT level was increased in SHRs given 10% fat diet compared to WKY rats, and the increased level was decreased by fimasartan treatment. In SHRs given 60% high-fat diet, the GPT level was more increased compared to WKY rats, and fimasartan treatment group showed the trend to lower the level (Figure 4(e)).

### 3.5. Determination of PPAR*δ*, AMPK, and PGC-1*α* Protein Levels, SDH Activity, and ATP Concentrations in Liver Tissue Samples Isolated from WKY Rats and SHRs

**Figure 5:**
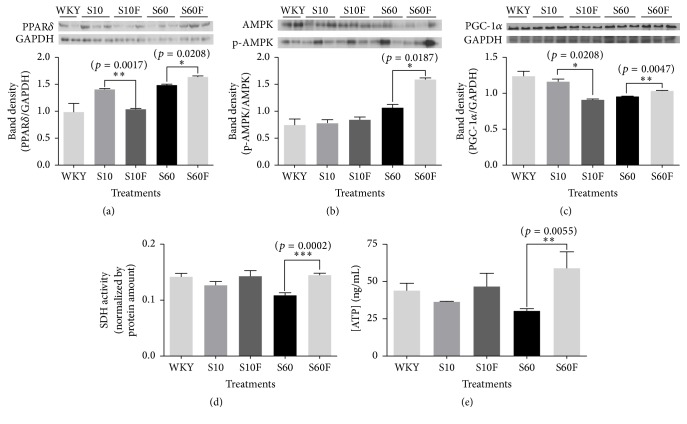
Peroxisome proliferator-activated receptor delta (PPAR*δ*), 5′ adenosine monophosphate-activated protein kinase (AMPK), and peroxisome proliferator-activated receptor gamma coactivator 1-alpha (PGC-1*α*) protein expressions, succinate dehydrogenase (SDH) activity, and adenosine triphosphate (ATP) concentration in liver tissues of in WKY rats and SHRs given high-fat diet and fimasartan. ((a), (b), and (c)) Protein extracts were electrophoresed in 10% polyacrylamide gel and blotted to nitrocellulose membrane. The nitrocellulose membrane was bound with primary and secondary antibodies sequentially, and then the chemiluminescent signal was exposed to X-ray film. The density of bands on X-ray film was analyzed with ImageJ program. (d) The SDH activity was assayed by enzymatic colorimetric method. (e) The concentration of ATP in liver extracts of WKY rats and SHRs was estimated with ATP ELISA assay kit. The results are expressed as means ± SEM (*n* = 3 or 6). Values were statistically analyzed by unpaired* t*-test. All experiments were repeated three times. Meaning of indications: WKY means normal control rat given normal diet containing 10% fat, S10 means SHRs given normal diet containing 10% fat, S10F means SHRs given normal diet containing 10% fat plus fimasartan, S60 means SHRs given high-fat diet containing 60% fat, and S60F means SHRs given high-fat diet containing 60% fat plus fimasartan.

PPAR*δ*, p-AMPK, and PGC-1*α* protein levels were shown to be significantly increased in the liver tissues isolated from SHRs fed high-fat diet and fimasartan compared with those in the samples isolated from control group fed high-fat diet (Figures 5(a)–5(c)). Succinate dehydrogenase is located in the inner mitochondrial membrane of the mammals, and it constitutes of both citric acid cycle and electron transport system. SDH activity and ATP level were shown to be decreased in liver tissue samples obtained from SHRs fed high-fat diet, in comparison with those measured in the samples obtained from WKY rats. However, following the fimasartan treatment, SDH activity and ATP concentration were significantly increased, compared with the untreated high-fat diet controls (Figures 5(d) and 5(e)).

### 3.6. H&E Staining and Adiponectin Concentration in Visceral Fat Tissues Isolated from WKY Rats and SHRs

As the size of adipocytes per unit square in fat tissues increases, the number of adipocytes decreases. Therefore, counting the number of adipocytes per unit area can represent the extent of lipid accumulation indirectly.

**Figure 6:**
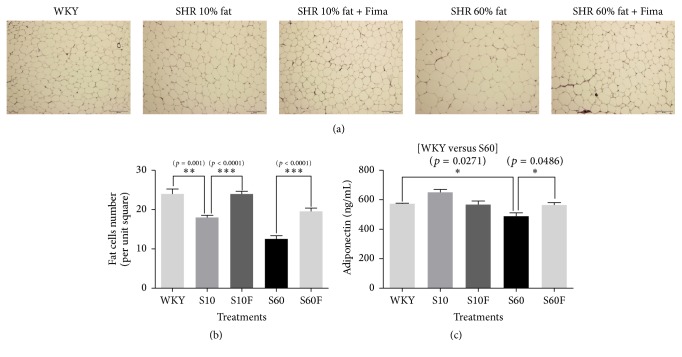
The number of adipocytes and adiponectin concentration in visceral fat tissues of WKY rats and SHRs given high-fat diet and fimasartan. (a) Fat tissue slides of WKY rats and SHRs were fixed and stained by hematoxylin and eosin reagents. Magnification is 100x. (b) The number of adipocytes per unit square (5461.06 *μ*m^2^) in slides stained by hematoxylin and eosin reagents. (c) The concentration of adiponectin in visceral fat extracts of WKY rats and SHRs was estimated with adiponectin colorimetric assay kit. The results are expressed as means ± SEM (*n* = 3 or 6). Values were statistically analyzed by unpaired* t*-test. All experiments were repeated three times. Meaning of indications: WKY means normal control rat given normal diet containing 10% fat, S10 means SHRs given normal diet containing 10% fat, S10F means SHRs given normal diet containing 10% fat plus fimasartan, S60 means SHRs given high-fat diet containing 60% fat, and S60F means SHRs given high-fat diet containing 60% fat plus fimasartan.

After H&E staining of visceral fat tissue slides, the number of adipocytes per unit square (5461.06 *μ*m^2^) was decreased in SHRs fed normal diet containing 10% fat compared to WKY rats, and the number is decreased more in SHRs given 60% high-fat diet. But fimasartan treatment increased the number of adipocytes in both SHRs fed 10% fat diet and SHRs given 60% high-fat diet (Figures 6(a) and 6(b)).

Adiponectin concentration in visceral fat tissues of SHRs given 60% high-fat diet was decreased compared to WKY rats; however, the concentration was increased by fimasartan (Figure 6(c)).

### 3.7. Expression Level of 11*β*-HSDH1, H&E Staining Intensity, and Triglyceride Concentration in Liver Tissue Samples Isolated from WKY Rats and SHRs

**Figure 7:**
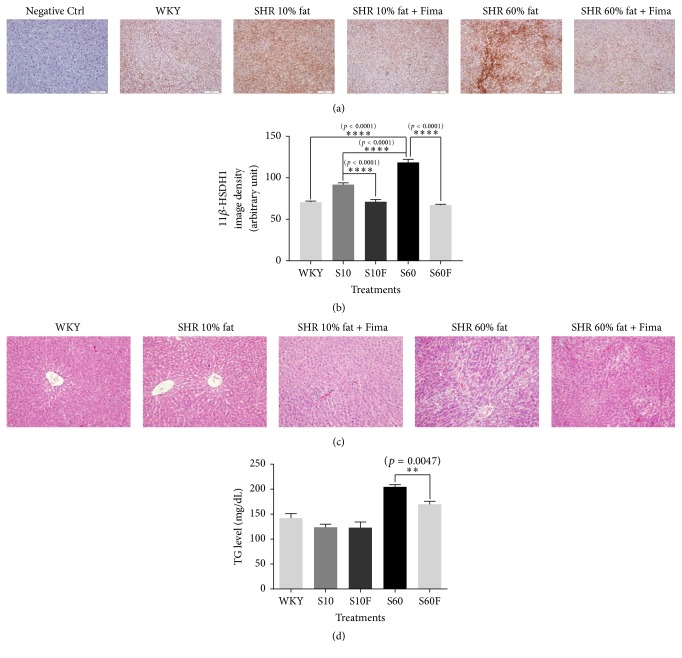
11 beta-hydroxysteroid dehydrogenase type 1 (11*β*-HSDH1) protein expression, hematoxylin and eosin staining, and the concentration of triglyceride in the liver tissue samples of WKY rats and SHRs given high-fat diet and fimasartan. (a) Liver tissue sections obtained from WKY rats and SHRs were fixed and stained by immunohistochemistry method for 11*β*-HSDH1. Magnification is 100x. (b) The densities for images were analyzed with the ImageJ program. (c) Liver tissue sections obtained from WKY rats and SHRs were fixed and stained with hematoxylin and eosin reagents. Magnification is 200x. (d) Triglyceride contents in liver extracts obtained from WKY rats and SHRs were determined with triglyceride colorimetric assay kit. The results are expressed as means ± SEM (*n* = 3 or 6). Values were statistically analyzed by unpaired* t*-test. All experiments were repeated three times. Meaning of indications: WKY means normal control rat given normal diet containing 10% fat, S10 means SHRs given normal diet containing 10% fat, S10F means SHRs given normal diet containing 10% fat plus fimasartan, S60 means SHRs given high-fat diet containing 60% fat, and S60F means SHRs given high-fat diet containing 60% fat plus fimasartan.

Fimasartan treatment was shown to decrease 11*β*-HSDH1 expression in liver tissue samples isolated from SHRs fed normal or high-fat diet (Figures 7(a) and 7(b)). Lipid content, shown to be increased in rats fed high-fat diet, was decreased by fimasartan treatment in SHRs' liver samples. However, no difference in lipid droplet content was observed between rats belonging to normal diet group and normal diet and fimasartan treatment SHRs group (Figure 7(c)). Triglyceride levels in liver tissues were significantly decreased following the treatment with fimasartan in SHRs fed high-fat diet (Figure 7(d)). This indicates that the hypolipidemic activity of fimasartan is exhibited only under hyperlipidemic condition.

## 4. Discussion

Aerobic exercise can lead to various beneficial changes in the body such as decreases of body weight, fat, and blood pressure and an increase of high-density lipoprotein (HDL) cholesterol level. Furthermore, it can have beneficial effects on the resting heart rate, physical fitness, and arterial stiffness in patients with metabolic syndrome [19]. In terms of molecular biology, exercise increases oxidative metabolism through the regulation of PGC-1*α*, citrate synthase, and SDH. And the effect of exercise is exerted by modulation of AMPK [20].

Metabolic syndrome is generally induced by nutritional excess and physical inactivity, and it can ultimately progress to several lifestyle-related diseases such as type 2 diabetes mellitus and cardiovascular disease. In a rat model expressing metabolic syndrome (SHR/NDmcr-*cp*), running exercise transformed the type of skeletal muscle fibers into highly oxidative form. In addition, the exercise increased PGC-1*α* mRNA expression and SDH activity in skeletal muscle [21].

PPAR*δ* and AMPK are key regulators of type 1 muscle fiber specification and endurance adaptations during exercise. In other words, the activation of PPAR*δ* and AMPK can induce the elevation of oxidative metabolism [20, 22].

Oxidative metabolism deficiencies represent one of the possible causes of NAFLD. For instance, hepatocytes of PGC-1*α* deficient mice have diminished fatty acid oxidation and mitochondrial respiration rates [23], and NAFLD is related to mitochondrial dysfunction [24].

Based on the results of these studies, we hypothesize that the beneficial effects of exercising are exerted through the increase in oxidative metabolism through the modulation of PGC-1*α*, citrate synthase, and SDH. These biomarkers are regulated by PPAR*δ* and AMPK, and, therefore, the activation of oxidative metabolism can be induced by stimulating PPAR*δ*, AMPK, and PGC-1*α* activity.

Here, we demonstrated that fimasartan increases PPAR*δ*, PGC-1*α*, p-AMPK, p-ACC, MCD, and MCAD levels in HepG2 cells treated with high fatty acid and that it decreases the expression levels of FAS, 11*β*-HSDH1, and TNF-*α*, which are increased by high fatty acid treatment. Furthermore, fimasartan increased the protein levels of PPAR*δ*, p-AMPK, and PGC-1*α* in the liver samples of SHRs fed high-fat diet, and the treatment with this compound elevated the activity of SDH and ATP level; also, it significantly decreased triglyceride levels in the liver samples obtained from these rats.

PPAR*δ* antagonist, GSK0660, was shown to inhibit fimasartan effects on p-AMPK and PGC-1*α* levels in HepG2 cells treated with high fatty acid. Additionally, it blocked the decrease in lipid content in HepG2 and 3T3-L1 cells treated with fimasartan.

These results suggest that fimasartan ameliorates lipid metabolism in the liver mainly through the activation of oxidative metabolism in hyperlipidemic condition, and the effect of fimasartan is primarily exerted by modulation of PPAR*δ*.

Several previous studies reported that 11*β*-HSDH1 is involved in the fatty acid metabolism. The inhibition of 11*β*-HSDH1 decreased weight gain, food intake, and fat pad weight in murine models of diet-induced obesity (DIO) and type 2 diabetes mellitus and in a mouse model of atherosclerosis, the apolipoprotein E (ApoE) knockout mouse [25]. Additionally, 11*β*-HSDH1 knockout mice were demonstrated to be resistant to hyperglycemia caused by obesity and stress, and they were shown to have low resistin and TNF-*α* levels and high PPAR*γ* and uncoupling protein 2 (UCP2) levels compared with these in the control [26]. In addition, PPAR (*α*, *γ*, and *δ*) pan agonist, bezafibrate, was shown to lead to a decrease in 11*β*-HSDH1 gene expression in liver and skeletal muscle tissues of db/db mice [27]. In this study, we demonstrated that fimasartan decreases the levels of 11*β*-HSDH1, FAS, and TNF-*α* which are increased under high fatty acid conditions and induces the expression of p-ACC.

The interrelation among adiponectin level, lipid metabolism, and metabolic diseases has been studied relatively well. Several examples are as follows: adiponectin treatment decreases the lipid accumulation in human macrophage cells [28], adiponectin regulates energy homeostasis through AMPK-ACC pathway [29], and adiponectin concentration in serum is lower in obese persons and patients with type 2 diabetes mellitus and NAFLD [30].

In previous studies, telmisartan induced the increase of serum adiponectin level and the change of morphology (decrease of cell size) of epididymal adipose cells in mice fed high-fat diet [31], irbesartan treatment decreased the GOT level and the diameter of adipocytes in apolipoprotein E knockout mice [32], and as hepatic steatosis increases, the activities of circulating aminotransferases such as GOT and GPT increase proportionally [33, 34]. Through these papers, it can be postulated that adiponectin is involved in the improvement of dyslipidemia, and the size of adipocytes represents one of healthy indexes for lipid metabolism.

In our study, fimasartan treatment increased the adiponectin level in visceral fat tissues from SHRs given 60% high-fat diet; however, in nontreated SHRs fed 60% high-fat diet, the level was lowered compared to WKY rats. Additionally, the size of visceral adipocytes became smaller by fimasartan treatment. Therefore, it is supposed that fimasartan ameliorates lipid metabolism via adiponectin regulation partly. Furthermore, in our research, the decreases of GOT and GPT activities by fimasartan are a definite additional evidence that fimasartan ameliorates NAFLD.

**Figure 8:**
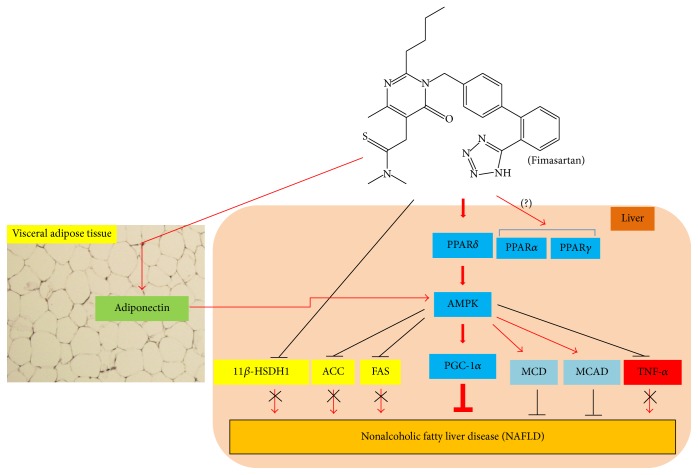
Schematic illustration for hypothetical reaction mechanisms of fimasartan in nonalcoholic fatty liver disease. Amelioration of nonalcoholic fatty liver disease by fimasartan is mainly accomplished with catabolic activation such as the sequential regulation of PPAR*δ*→ AMPK → PGC-1*α*. In addition,* Aster glehni *lowers triglyceride accumulation in liver by inhibiting 11*β*-HSDH1, ACC, FAS, and TNF-*α*; also, its effect is additionally exerted by activating adiponectin, MCD, and MCAD. Meaning of symbols: arrow means activation, up and horizontal line means inhibition, and X means blocking.

In conclusion, fimasartan may ameliorate NAFLD mainly through the activation of oxidative metabolism represented by PPAR*δ*-AMPK-PGC-1*α* pathway, and its effect may be additionally derived from the inhibition of fatty acid synthesis (11*β*-HSDH1, FAS, and ACC) and inflammation (TNF*α*), also the elevation of fatty acid oxidation (MCD and MCAD) and adiponectin level (Figure 8).

In our study, the mRNA levels of PPAR*α* and PPAR*γ* were not changed by fimasartan treatment (Figure 1), and PPAR*α* protein expression was decreased by fimasartan in HepG2 cells (additional Figure 1 in Supplementary Material available online at https://doi.org/10.1155/2017/8048720), and its level was not changed with fimasartan in liver tissues of SHRs given 60% high-fat diet (additional Figure 2). Therefore, it can be suggested that PPAR*δ* is the main target of fimasartan in liver of hyperlipidemic and hypertensive conditions. But this assumption should be strictly proven through additional studies including more experiments about the effects of fimasartan on fatty acid oxidation in following study.

## Supplementary Material

TRIzol^®^ reagent was purchased from Invitrogen (Grand Island, NY, USA). Power cDNA Synthesis kits and Maxime^™^ PCR PreMix kits were purchased from Intron Biotechnology (Seongnam-si, Gyeonggi-do, Korea). Primary antibody for peroxisome proliferator-activated receptor alpha (PPARα) was supplied from Abcam (Cambridge, UK).

## References

[1] Duseja A., Nanda M., Das A., Das R., Bhansali A., Chawla Y. (2004). Prevalence of obesity, diabetes mellitus and hyperlipidaemia in patients with cryptogenic liver cirrhosis. *Tropical Gastroenterology*.

[2] Liangpunsakul S., Chalasani N. (2003). Treatment of nonalcoholic fatty liver disease. *Current Treatment Options in Gastroenterology*.

[3] Kneeman J. M., Misdraji J., Corey K. E. (2012). Secondary causes of nonalcoholic fatty liver disease. *Therapeutic Advances in Gastroenterology*.

[4] Farrell G. C. (2003). Non-alcoholic steatohepatitis: what is it, and why is it important in the Asia-Pacific region?. *Journal of Gastroenterology and Hepatology (Australia)*.

[5] Cong W.-N., Tao R.-Y., Tian J.-Y., Liu G.-T., Ye F. (2008). The establishment of a novel non-alcoholic steatohepatitis model accompanied with obesity and insulin resistance in mice. *Life Sciences*.

[6] Kiyici M., Gulten M., Gurel S. (2003). Ursodeoxycholic acid and atorvastatin in the treatment of nonalcoholic steatohepatitis. *Canadian Journal of Gastroenterology*.

[7] Eslami L., Merat S., Malekzadeh R., Nasseri-Moghaddam S., Aramin H. (2013). Statins for non-alcoholic fatty liver disease and non-alcoholic steatohepatitis. *The Cochrane Database of Systematic Reviews*.

[8] Kudo H., Yata Y., Takahara T. (2009). Telmisartan attenuates progression of steatohepatitis in mice: role of hepatic macrophage infiltration and effects on adipose tissue. *Liver International*.

[9] Lee H. W., Lim M.-S., Seong S. J. (2011). Effect of age on the pharmacokinetics of fimasartan (BR-A-657). *Expert Opinion on Drug Metabolism and Toxicology*.

[10] Kim C. O., Lee H. W., Oh E. S. (2013). Influence of hepatic dysfunction on the pharmacokinetics and safety of fimasartan. *Journal of Cardiovascular Pharmacology*.

[11] Berger J., Moller D. E. (2002). The mechanisms of action of PPARs. *Annual Review of Medicine*.

[12] Feige J. N., Gelman L., Michalik L., Desvergne B., Wahli W. (2006). From molecular action to physiological outputs: peroxisome proliferator-activated receptors are nuclear receptors at the crossroads of key cellular functions. *Progress in Lipid Research*.

[13] Beauloye C., Bertrand L., Horman S., Hue L. (2011). AMPK activation, a preventive therapeutic target in the transition from cardiac injury to heart failure. *Cardiovascular Research*.

[14] Shirwany N. A., Zou M.-H. (2014). AMPK: a cellular metabolic and redox sensor. A minireview. *Frontiers in Bioscience—Landmark*.

[15] National Center for Biotechnology Information FASN fatty acid synthase [Homo sapiens (human)]. http://www.ncbi.nlm.nih.gov/gene?Db=gene&Cmd=ShowDetailView&TermToSearch=2194.

[16] Seckl J. R., Walker B. R. (2004). 11*β*-hydroxysteroid dehydrogenase type 1 as a modulator of glucocorticoid action: from metabolism to memory. *Trends in Endocrinology and Metabolism*.

[17] Sambandam N., Steinmetz M., Chu A., Altarejos J. Y., Dyck J. R. B., Lopaschuk G. D. (2004). Malonyl-CoA decarboxylase (MCD) is differentially regulated in subcellular compartments by 5′AMP-activated protein kinase (AMPK) Studies using H9c2 cells overexpressing MCD and AMPK by adenoviral gene transfer technique. *European Journal of Biochemistry*.

[18] Cao S., Zhou Y., Xu P. (2013). Berberine metabolites exhibit triglyceride-lowering effects via activation of AMP-activated protein kinase in Hep G2 cells. *Journal of Ethnopharmacology*.

[19] Kang S.-J., Kim E.-H., Ko K.-J. (2016). Effects of aerobic exercise on the resting heart rate, physical fitness, and arterial stiffness of female patients with metabolic syndrome. *Journal of Physical Therapy Science*.

[20] Röckl K. S. C., Hirshman M. F., Brandauer J., Fujii N., Witters L. A., Goodyear L. J. (2007). Skeletal muscle adaptation to exercise training: AMP-activated protein kinase mediates muscle fiber type shift. *Diabetes*.

[21] Nagatomo F., Fujino H., Kondo H. (2012). The effects of running exercise on oxidative capacity and PGC-1*α* mRNA levels in the soleus muscle of rats with metabolic syndrome. *Journal of Physiological Sciences*.

[22] Narkar V. A., Downes M., Yu R. T. (2008). AMPK and PPAR*δ* agonists are exercise mimetics. *Cell*.

[23] Finck B. N., Kelly D. P. (2006). PGC-1 coactivators: inducible regulators of energy metabolism in health and disease. *Journal of Clinical Investigation*.

[24] Wei Y., Rector R. S., Thyfault J. P., Ibdah J. A. (2008). Nonalcoholic fatty liver disease and mitochondrial dysfunction. *World Journal of Gastroenterology*.

[25] Hermanowski-Vosatka A., Balkovec J. M., Cheng K. (2005). 11*β*-HSD1 inhibition ameliorates metabolic syndrome and prevents progression of atherosclerosis in mice. *The Journal of Experimental Medicine*.

[26] Morton N. M., Paterson J. M., Masuzaki H. (2004). Novel adipose tissue-mediated resistance to diet-induced visceral obesity in 11*β*-hydroxysteroid dehydrogenase Type 1-deficient mice. *Diabetes*.

[27] Nakano S., Inada Y., Masuzaki H. (2007). Bezafibrate regulates the expression and enzyme activity of 11*β*-hydroxysteroid dehydrogenase type 1 in murine adipose tissue and 3T3-L1 adipocytes. *American Journal of Physiology—Endocrinology and Metabolism*.

[28] Ouchi N., Kihara S., Arita Y. (2001). Adipocyte-derived plasma protein, adiponectin, suppresses lipid accumulation and class A scavenger receptor expression in human monocyte-derived macrophages. *Circulation*.

[29] Wen J.-P., Liu C.-E., Hu Y.-T., Chen G., Lin L.-X. (2010). Globular adiponectin regulates energy homeostasis through AMP-activated protein kinase-acetyl-CoA carboxylase (AMPK/ACC) pathway in the hypothalamus. *Molecular and Cellular Biochemistry*.

[30] Petta S., Gastaldelli A., Rebelos E. (2016). Pathophysiology of non alcoholic fatty liver disease. *International Journal of Molecular Sciences*.

[31] Araki K., Masaki T., Katsuragi I., Tanaka K., Kakuma T., Yoshimatsu H. (2006). Telmisartan prevents obesity and increases the expression of uncoupling protein 1 in diet-induced obese mice. *Hypertension*.

[32] Chatterjee A., Kusunoki H., Taniyama Y., Rakugi H., Morishita R. (2013). Improvement of metabolic syndrome by irbesartan via the PPAR*γ*/HGF pathway in apolipoprotein E knockout mice. *Biomedical Reports*.

[33] Sookoian S., Castaño G. O., Scian R. (2016). Serum aminotransferases in nonalcoholic fatty liver disease are a signature of liver metabolic perturbations at the amino acid and Krebs cycle level. *The American Journal of Clinical Nutrition*.

[34] Unalp-Arida A., Ruhl C. E. (2016). Noninvasive fatty liver markers predict liver disease mortality in the U.S. population. *Hepatology*.

