# Supplementary material for: Fimasartan Ameliorates Nonalcoholic Fatty Liver Disease through PPARδ Regulation in Hyperlipidemic and Hypertensive Conditions
Source: PPAR Res. 2017 Mar 13;2017:8048720. doi: 10.1155/2017/8048720 (PMC5366769; doi:10.1155/2017/8048720)

Additional Figure legends

Additional Fig. 1 Peroxisome proliferator-activated receptor alpha (PPARα) in HepG2 cells treated with high fatty acid and fimasartan.

Protein extracts were electrophoresed in 10% poly acrylamide gel and blotted to nitrocellulose membrane. The nitrocellulose membrane was bound with primary and secondary antibodies sequentially, and then the chemiluminescent signal was exposed to X-ray film. The density of bands on X-ray film was analyzed with Image J program. The results are expressed as means ± SEM (n=3). Values were statistically analyzed by unpaired *t*-test. All experiments were repeated three times.

Additional Fig. 2 Peroxisome proliferator-activated receptor alpha (PPARα) in liver tissues of SHRs administered with high fat diet and fimasartan.

Protein extracts were electrophoresed in 10% poly acrylamide gel and blotted to nitrocellulose membrane. The nitrocellulose membrane was bound with primary and secondary antibodies sequentially, and then the chemiluminescent signal was exposed to X-ray film. The density of bands on X-ray film was analyzed with Image J program. The results are expressed as means ± SEM (n=6). Values were statistically analyzed by unpaired *t*-test. All experiments were repeated three times.

Meaning of indications: S60 means SHR administered with high fat diet containing 60% fat , and S60F means SHR administered with high fat diet containing 60% fat plus fimasartan.

Additional Fig. 1

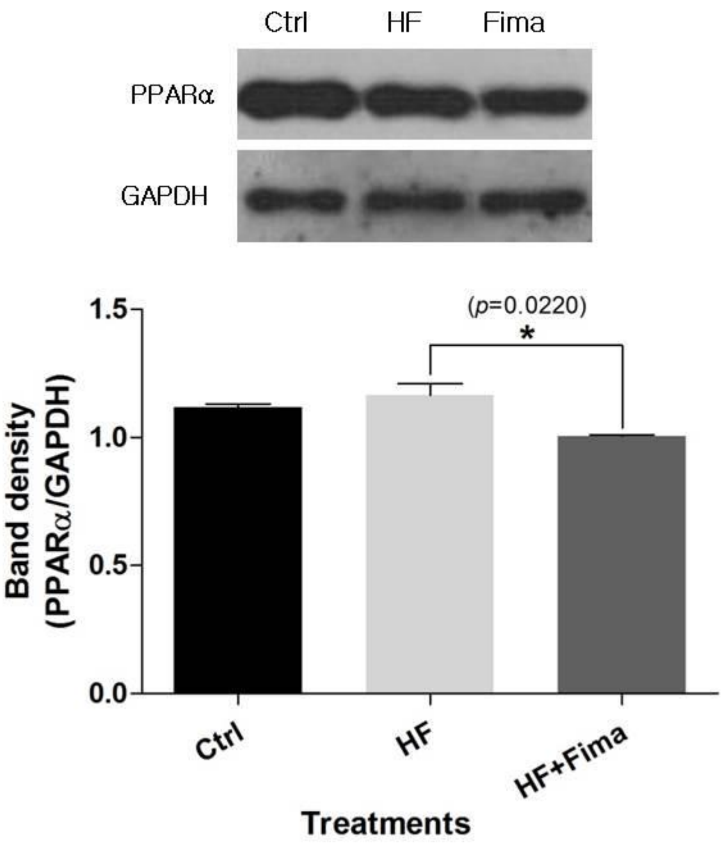

Additional Fig. 2

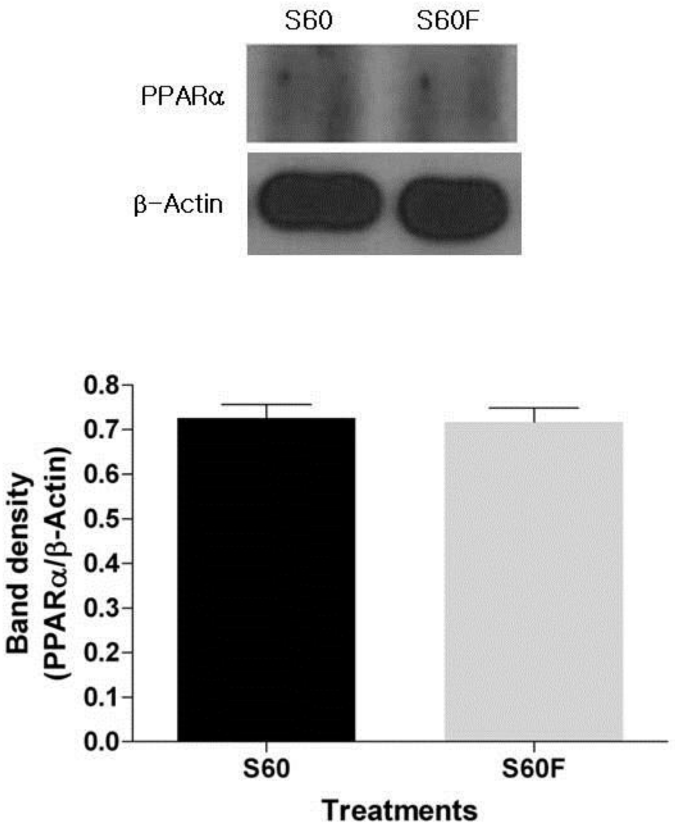

Supplement: Supplementary file 1 — TRIzol® reagent was purchased from Invitrogen (Grand Island, NY, USA). Power cDNA Synthesis kits and Maxime™ PCR PreMix kits were purchased from Intron Biotechnology (Seongnam-si, Gyeonggi-do, Korea). Primary antibody for peroxisome proliferator-activated receptor alpha (PPARα) was supplied from Abcam (Cambridge, UK). [file 8048720.f1.pdf]
